# Simple and rapid direct cloning and heterologous expression of natural product biosynthetic gene cluster in *Bacillus subtilis* via Red/ET recombineering

**DOI:** 10.1038/srep34623

**Published:** 2016-09-30

**Authors:** Qingshu Liu, Qiyao Shen, Xiaoying Bian, Hanna Chen, Jun Fu, Hailong Wang, Ping Lei, Zhaohui Guo, Wu Chen, Dingjun Li, Youming Zhang

**Affiliations:** 1 College of Plant Protection, Hunan Agricultural University, Changsha, 410128, People’s Republic of China; 2 Shandong University–Helmholtz Institute of Biotechnology, State Key Laboratory of Microbial Technology, School of Life Science, Shandong University, Jinan, 250100, People’s Republic of China; 3 Hunan Institute of Microbiology, Changsha, 410009, People’s Republic of China; 4 Hunan Business College, Changsha, 410205, People’s Republic of China

## Abstract

Heterologous expression of biosynthetic pathways is an important way to research and discover microbial natural products. *Bacillus subtilis* is a suitable host for the heterologous production of natural products from bacilli and related Firmicutes. Existing technologies for heterologous expression of large biosynthetic gene clusters in *B. subtilis* are complicated. Herein, we present a simple and rapid strategy for direct cloning based heterologous expression of biosynthetic pathways in *B. subtilis* via Red/ET recombineering, using a 5.2 kb specific direct cloning vector carrying homologous sequences to the *amyE* gene in *B. subtilis* and CcdB counterselection marker. Using a two-step procedure, two large biosynthetic pathways for edeine (48.3 kb) and bacillomycin (37.2 kb) from *Brevibacillus brevis* X23 and *B. amyloliquefaciens* FZB42, respectively, were directly cloned and subsequently integrated into the chromosome of *B. subtilis* within one week. The gene cluster for bacillomycin was successfully expressed in the heterologous host, although edeine production was not detectable. Compared with similar technologies, this method offers a simpler and more feasible system for the discovery of natural products from bacilli and related genera.

Microorganisms produce a large number of secondary metabolites, the main resource for pharmaceuticals and bio-pesticides^1^,^2^,^3^,^4^. Many of these natural products belong to the polyketide and nonribosomal peptide families synthesized by large multienzyme megasynthetases, known as polyketide synthases (PKSs) and nonribosomal peptide synthetases (NRPSs), respectively^5^,^6^,^7^. Occasionally, the transfer of a secondary metabolite pathway from the original producing microorganism into a suitable heterologous host, that is, heterologous expression, is warranted for the following purposes: to demonstrate the gene cluster required for the biosynthesis of a compound; to increase the yield of a compound; to discover microbial natural products, which are difficult to culture or not genetically amenable; or to generate natural product derivatives or unnatural products by modification of biosynthetic pathways in a genetically amenable host^8^,^9^.

Bacilli is one of the largest resources of natural products, from which numerous secondary metabolite biosynthetic pathways have been detected^10^,^11^,^12^. The increasing number of published genome sequences of bacilli strains has revealed that a large number of biosynthetic pathways are cryptic in bacilli, particularly for strains not belonging to *Bacillus* genera^12^. These bacteria are generally difficult to genetically manipulate, thereby impeding the discovery of novel natural products from these microbes by genome mining^13^. However, the cloning and heterologous expression of these cryptic gene clusters in an amenable host has been proven to be a feasible approach to uncover natural products from genetically hard-to-handle bacteria^8^,^9^,^14^. The selection of a suitable heterologous host as well as efficient cloning and transferring large gene clusters is the key issue for successful heterologous expression^8^. *Bacillus subtilis* is the best host for the heterologous expression of the secondary metabolite biosynthetic pathways from *Bacillus* and related genera. First, *B. subtilis* is a well-studied non-pathogenic bacteria with excellent fermentation characteristics^15^,^16^. Second, *B. subtilis* possesses functional and genetic background similar to its related species and genera for the biosynthesis of natural products and their required precursors. Third, *B. subtilis* has sophisticated and simple genetic operation approaches and tools, particularly for the integration of large foreign DNA fragments into the chromosome by natural transformation, with a capacity of more than 3 Mb^17^,^18^. Several secondary metabolite biosynthetic pathways have been successfully expressed in *B. subtilis*, for example, bacitracin biosynthetic gene cluster from *B. licheniformis*^19^, nisin biosynthetic gene cluster from Lactococcus lactis^20^, polymyxin biosynthetic gene cluster from *Paenibacillus polymyxa*^21^, enniatin biosynthetic gene cluster from *Fusarium*.sp^22^, amicoumacin biosynthetic gene cluster from *B. subtilis*^14^, and 6 -deoxyerythronolide B biosynthetic gene cluster from *Saccharopolyspora erythraea*^23^.

However, obtaining secondary metabolite biosynthetic gene clusters is challenging, as these gene clusters are typically large, ranging from10 to 100 kb. Conventionally, fosmid or cosmid genomic libraries have been constructed to obtain the gene clusters of interest; however screening the correct clones is an extremely time-consuming and laborious task^24^,^25^. Recently, simple technologies have been developed to directly clone large biosynthetic gene clusters from genomic DNA, such as Gibson assembly^26^, transformation-associated recombination (TAR)^27^, φ BT1 integrase-mediated direct cloning^28^, oriT-directed capture^29^, Cas9-Assisted Targeting of Chromosome segments (CATCH)^30^, and Red/ET recombineering^31^. Direct cloning via Red/ET recombineering^32^,^33^ is based on *in vivo* linear plus linear homologous recombination (LLHR) in *E. coli* mediated by the prophage recombinases RecE and RecT, representing a powerful method for the cloning and genetic engineering of natural product biosynthetic pathways. To date, several gene clusters have been cloned and heterologously expressed using this method, including the luminmycin^31^,^34^, luminmide^31^, syringolin^35^, glidobactin^34^, colibactin^36^, sevadicin^37^, and salinomycin^38^ gene clusters.

Linear cloning vector for direct cloning in Red/ET recombineering comprises only two elements, the plasmid replication origin and antibiotic selection marker maintained in *E. coli*^29^, lacking maintenance or transfer elements for other heterologous hosts. Thus, several steps for further modifications were required to add heterologous expression elements, such as a suitable promoter, appropriate antibiotic selection marker and chromosomal integration elements, to the cloned gene cluster^38^. Therefore, in the present study, we constructed a specific cloning vector for direct cloning carrying homologous sequence to *B. subtilis* and established a simple and rapid strategy for the direct cloning of natural product biosynthesis gene clusters and expression in *B. subtilis* via Red/ET recombineering. The recombinant plasmid that contains the cloned gene cluster can be directly integrated into the chromosome of *B. subtilis* without further modification. For proof of principal, two NPRS gene clusters, the edeine^39^ biosynthetic pathway (48.9 kb) from a biocontrol bacteria *Brevibacillus brevis* X23^40^ and the bacillomycin^41^ biosynthetic pathway (37.2 kb) from *B. amyloliquefaciens* FZB42^42^ were directly cloned using this vector and each one was integrated into chromosome of *B. subtilis* within one week. The results showed bacillomycin has been successfully produced in the heterologous host. This work will further facilitate the heterologous expression of natural product biosynthetic pathways in *B. subtilis*.

## Results

### Repair of the *sfp* gene in *B. subtilis* 1A751 to construct a functional host for NRPS and PKS

**Figure 1:**
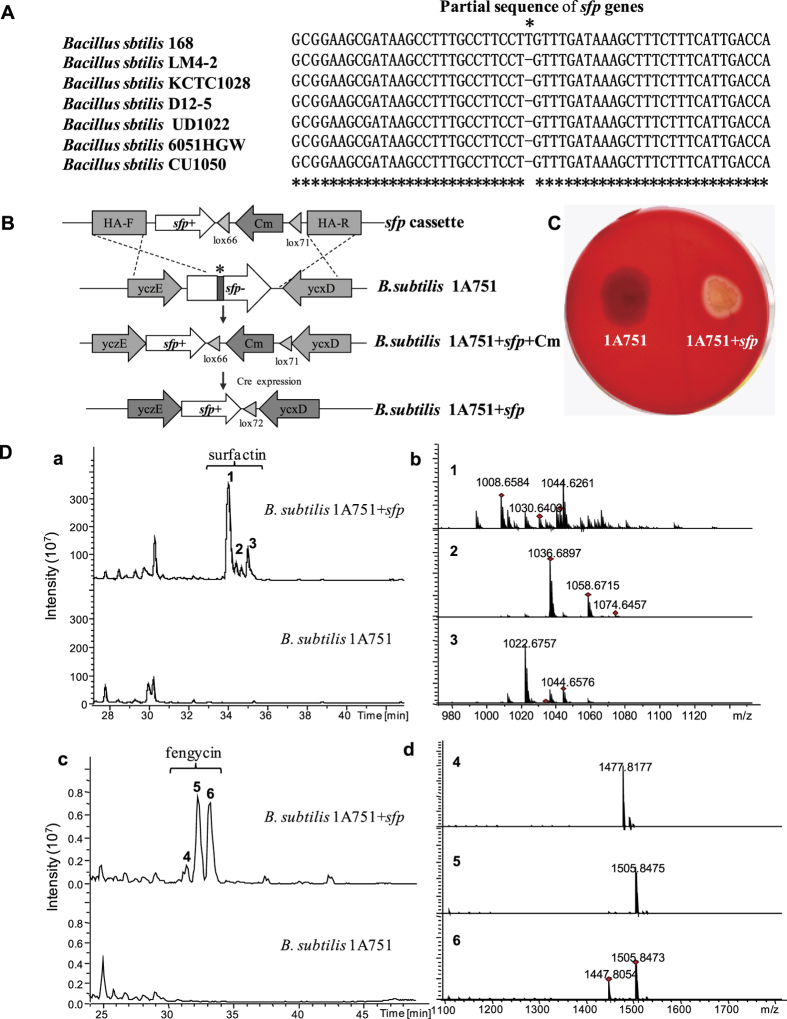
Construction of a heterologous expression host for NPRS/PKS gene cluster based on *B. subtilis* 1A751. (**A**) Detection mutation of the *sfp* gene in *B. subtilis* 168 (original strain of *B. subtilis* 1A751) after alignment with the intact *sfp* gene from six different *B. subtilis* strains. (**B**) Scheme for repairing the insertional mutation of the *sfp* gene in *B. subtilis* 1A751. (**C**) Haemolytic analysis of *B. subtilis* 1A751 (1A751) and transformants of the *sfp*-repaired derivative *B. subtilis* 1A751 + *sfp* (1A751 + *sfp*). (**D**) Analysis of surfactin and fengycin production in *B. subtilis* 1A751 + *sfp*. (**a**) HPLC-MS analysis of surfactin production in *B. subtilis* 1A751 + *sfp*. (**b**) MS spectra of three surfactin peaks(1, 2, and 3). Compound 1 indicates C13-surfactin (m/z 1008.6584 [M + H]^+^and 1030.6408 [M + Na]^+^), Compound 2 indicates C15-surfactin (m/z 1036.6897 [M + H]^+^and 1058.6715 [M + Na]^+^), Compound 3 indicates C14-surfactin (m/z 1022.6757 [M + H]^+^and 1044.6576 [M + Na]^+^), (**c**) HPLC-MS analysis of fengycin produced from *B. subtilis* 1A751 + *sfp*. (**d**) MS spectra of the three peaks of fengycin produced from *B. subtilis* 1A751 + *sfp*. Compound 4 indicates Ala-6-C17 fengycin (m/z 1477.8177 [M + H]^+^), and compound 5 indicates Val-6-C17 fengycin (m/z 1505.8 [M + H]^+^). Peak 6 is a mixture of Ala-6-C17 fengycin and Val-6-C17 fengycin.

*B. subtilis* 1A751^43^, a two protease-deficient derivative of *B. subtilis*168^18^, has a clear genetic background, sophisticated genetic manipulation method and lower protease activity, making this bacterium to be a good candidate as a heterologous host. Although *B. subtilis* 168 and its derivative, *B. subtilis* 1A751, have several biosynthetic gene clusters for PKSs and NRPSs in the chromosome^44^, such as surfactin and fengycin^44^, these strains cannot produce these secondary metabolites, as the *sfp* gene encoding for 4′phosphopantetheinyl transferase (PPTase), an essential enzyme for the production of both PKS and NRPS^45^, is mutated and dysfuntional^45^. Sequence alignment with the functional *sfp* genes from other *B. subtilis* strains, including *B. subtilis* LM4-2(gb: CP011101.1), *B. subtilis* KCTC1028 (gb: CP011115.1), *B. subtilis* D12-5 (gb: CP014858.1), *B. subtilis* UD1022 (gb: CP011534.1), *B. subtilis* 6051HGW (gb: CP003329.1), and *B. subtilis* CU1050 (gb: CP014166.1), showed a single-DNA-base T insertional mutation resulting in a frameshift at 105 bp downstream from the ATG start codon in the *sfp* gene of *B. subtilis* 168 (Fig. 1A). To demonstrate whether this insertional mutation was solely responsible for *sfp* gene inactivation in *B. subtilis* 1A751, we deleted this base-pair insertion using *in situ* integration of a repaired *sfp* gene via the repair vector pBR322-Amp-HAF-sfp-lox-cm-HAR and subsequently excised the chloramphenicol resistance gene (Cm) using the Cre-lox system^46^, as illustrated in Fig. S1 and Fig. 1B. After verification by sequencing, the correct transformants, *B. subtilis* 1A751 + *sfp*, were subjected to haemolytic activity analyses by blood agar methods^47^. After a 2-day incubation at 37 °C, a colourless, transparent ring around the colony of the *sfp* gene repaired strain, *B. subtilis* 1A751 + *sfp*, was detected as a qualitative indicator of the production of surfactin^47^, while no transparent ring was detected around the colony of *B. subtilis* 1A751 (Fig. 1C). Subsequently, LC-MS detection was conducted to further verify the production of surfactin and other secondary metabolites in *B. subtilis* 1A751 + *sfp*. Subsequently, a differential analysis of the metabolite profiles between *B. subtilis* 1A751 and *B. subtilis* 1A751 + *sfp* were performed using HPLC-MS. Three major surfactin analogues, C13-surfactin (m/z 1008.6 [M + H]^+^), C14-surfactin (m/z 1022.7 [M + H]^+^), and C15-surfactin (m/z 1036.7 [M + H]^+^) (Fig. 1Da/Db) and two major fengycin analogues, Ala-6-C17 fengycin (m/z 1477.8 [M + H]^+^) and Val-6-C17 fengycin (m/z 1505.8 [M + H]^+^) (Fig. 1Da/Db) were detected in *B. subtilis* 1A751 + *sfp*. Instead of transferring a foreign *sfp* gene^14^,^21^, this study is the first to report the *in situ* repair of a point mutation in the *sfp* gene of the *B. subtilis* 168 derivative, generating a PKS/NPRS producer strain. Furthermore, this strain could also serve as a heterologous host for the expression of PKS/NRPS gene clusters from bacilli.

### Construction of a cloning vector for direct cloning and heterologous expression in *B. subtilis*

To construct a specific cloning vector for direct cloning containing elements for gene cluster horizontal transfer to *B. subtilis*, five fragments (backbone of p15A, amyEF, Amp-ccdB, Spect, and amyER) with 50-bp overlapping sequences in both terminal were co-transformed into *E. coli* GB05dir-gyrA462^48^, a CcdB-resistant *E. coli* strain containing RecE and RecT recombinases, to generate the cloning vector p15A-amyEF-Amp-ccdB-Spect-amyER by Red/ET quintuple LLHR, as illustrated in Fig. S2. The resulting direct cloning vector comprised four elements, a p15A origin for plasmid propagation in *E. coli*, a versatile spectinomycin resistance gene for selection in both *E. coli* and *B. subtilis*, two homologous arms (HA) upstream and downstream of the *amy*E gene (amyEF and amyER) of *B. subtilis* for the specific integration of cloned gene clusters into the chromosome of *B. subtilis* 1A751 + *sfp* in the *amy*E gene loci, and a counterselection marker gene *ccdB*^48^ to reduce the background of the original vector during PCR generation of the linear cloning vector. After restriction endonuclease digestion analysis and sequencing, we validated the function of the toxic protein CcdB as previously described^44^. To this end, we transformed the vector p15A-amyEF-Amp-ccdB-Spect-amyER into *E. coli* GB-dir, a CcdB-sensitive strain, and *E. coli* GB05dir-gyrA462^48^, a CcdB-resistant *E. coli* strain. After recovery, 10 μl of culture were plated onto LB plates supplemented with spectinomycin. No transformants were detected on the plates containing the vector transformed into the CcdB-sensitive strain, while numerous of transformants were detected on plates containing the vector transformed into the CcdB-resistant *E. coli* strain. These results suggested that the ccdB gene in the vector p15A-amyEF-Amp-ccdB-Spect-amyER is functional to reduce the background of the original vector.

### Direct cloning of biosynthesis gene clusters of natural products

**Figure 2:**
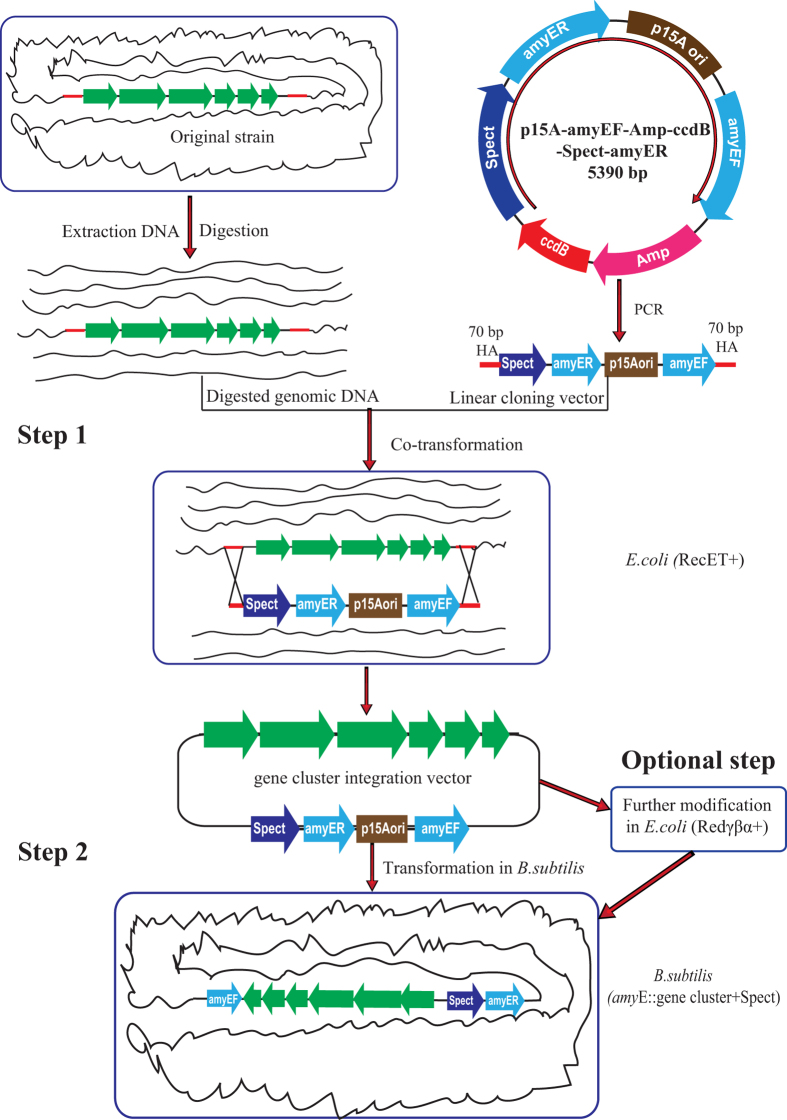
General scheme of direct cloning and heterologous expression of PKS/NRPS gene clusters in *B. subtilis* via Red/ET recombineering. In step 1, the digested genomic DNA of the producer strain and linear cloning vector carrying the 70-bp homologous arm with target gene cluster were co-transferred in *E. coli* GB05-dir to directly clone the target gene cluster into the integration vector by RecET-mediated LLHR. In an optional step (if necessary), some of the cloned gene clusters were further modified by Redγβα-mediated LCHR(linear plus circular homologous recombination), for example, to delete homologous region or key genes, exchange promoters, and add extra genes or elements. In step 2, if the cloned gene clusters did not require further modification, the cloned gene cluster would be directly integrated into the *amy*E gene locus of the heterologous host *B. subtilis* by natural competence transformation to express the candidate secondary metabolites.

To demonstrate the feasibility of the improved cloning vector, we cloned and heterologously expressed two desired NPRS gene clusters: the biosynthetic pathway for edeine (*ede*, 48.9 kb), an atypical cationic peptide with broad-spectrum antimicrobial activity^37^ from the biocontrol bacteria *Brevibacillus brevis* X23^38^, and the biosynthetic pathway for bacillomycin (bmy, 37.2 kb), an antifungal lipopeptide from *B. amyloliquefaciens* FZB42^40^, according to the two-step procedure described in Fig. 2. Using the cloning vector p15A-amyEF-Amp-ccdB-Spect-amyER as the template, we designed two pairs of primers to generate linear cloning vectors (p15A-amyEF-Spect-amyER) carrying the terminal 70-bp homology arms of *ede* and *bmy* gene clusters, respectively, using PCR. The 5′ and 3′ homology arms were selected at the location of suitable restriction enzyme sites flanking the gene clusters. Using one step LLHR, these two biosynthetic gene clusters were successfully cloned into the cloning vectors, with the efficiency of 2/24 and 3/24, respectively. The resulting recombinant plasmids containing the *ede* or *bmy* gene clusters were designed as p15A-amyEF-Spect-amyER-ede and p15A-amyEF-Spect-amyER-bmy. The correct clones were verified by restriction enzyme analysis (Fig. S3A/B). Consistent with the close genetic relationship between the producer strain and heterologous host stain, all of the cloned gene clusters were under the control of their original promoters. The *ede* gene cluster of the recombinant plasmid p15A-amyEF-Spect-amyER-ede can be directly used to transfer into *B. subtilis* without further modification. Another gene cluster-containing plasmid, p15A-amyEF-Spect-amyER-bmy, contains a 1.5 kb redundant region downstream from the bacillomycin gene cluster, which was highly homologous to the host strain *B. subtilis* 1A751 + *sfp*. We knocked out this region by linear plus circular homologous recombination (LCHR) mediated by the λ phage recombinase Redαβγ using a lox71-cm-lox66 cassette and excised the antibiotic marker by a Cre-loxP systems to ensure the specific integration of the cloned gene cluster into the *amy*E gene locus of *B. subtilis* 1A751 + *sfp*, generating the recombinant plasmid p15A-amyEF-Spect-amyER-bmy2, followed by verification by restriction enzyme analysis (Fig. S3B).

### Heterologous expression of the bacillomycin biosynthetic gene cluster

**Figure 3:**
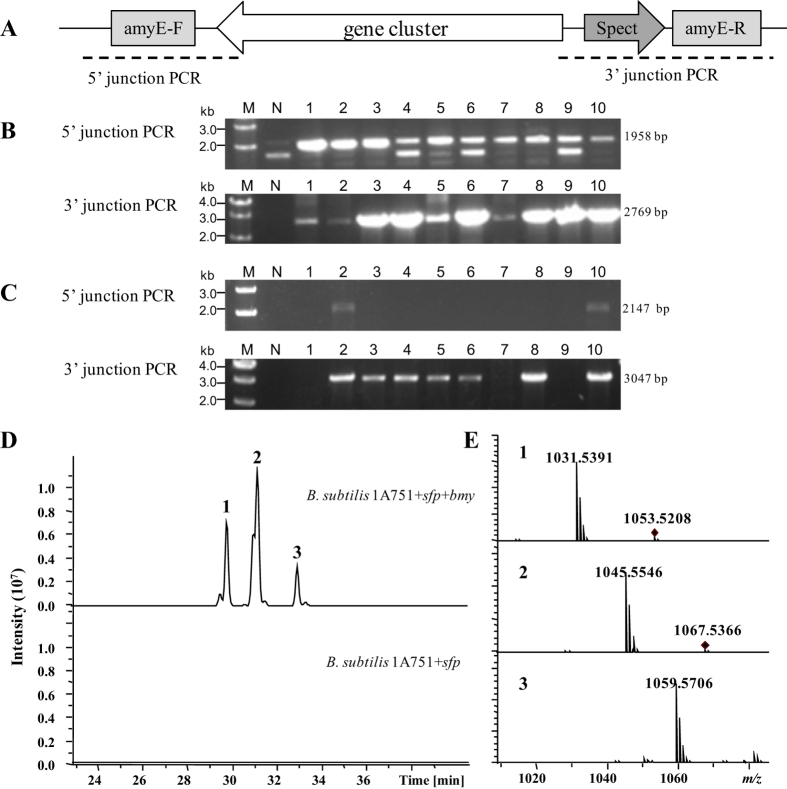
Verification of the correct integration of the two gene clusters and bacillomycin production in *B. subtilis* 1A751 + *sfp*. (**A**) Schematic presentation of gene cluster integration in *B. subtilis* 1A751 + *sfp* and PCR setup. (**B**). Detection correct clones of insertion of *ede* gene cluster in *B. subtilis* 1A751 + *sfp* by 5′ and 3′ junction PCR. This figure present the method of screening the correct transformants. The fragment of 5′ junction PCR indicates that the gene cluster was correctly integrated in the amyE-F locus. And the fragment of 3′ junction PCR indicates that the gene cluster was correctly integrated in the amyE-R locus. The correct transformants should have both 5′ junction PCR fragment and 3′ junction PCR fragment. Lane M is the Takara 1kb ladder. Lane N is the host stain as negative control. Lanes 1–10 are recombinants. The predicted size of the PCR products of 5′ and 3′ junction PCR are 1958 bp and 2769 bp, respectively. All ten clones are correct transformant resulting from double crossover. (**C**). Detection of the correct clones of *bmy* gene cluster in *B. subtilis* 1A751 + *sfp* by 5′ and 3′ junction PCR. The predicted size of the PCR products of 5′ and 3′ junction PCR are 2147 bp and 3047 bp, respectively. Clone 2 and clone 10 are correct transformant resulting from double crossover. Clones 3/4/5/6/8 might result from single crossover. (**D**). HPLC-MS analysis of bacillomycin production in *B. subtilis* 1A751 + *sfp*. (**E**). MS spectra of compounds 1, 2 and 3.Compound 1 indicates C14-bacillomycin D (m/z 1031.5391 [M + H]^+^and 1053.5208 [M + Na]^+^). Compound 2 indicates C15-bacillomycin D (m/z 1045.5546 [M + H]^+^and 1067.5366 [M + Na]^+^). Compound 3 indicates C16-bacillomycin D (m/z 1059.5706 [M + H]^+^).

The recombinant plasmids p15A-amyEF-Spect-amyER-ede and p15A-amyEF-Spect-amyER-bmy2 were transferred into *B. subtilis* 1A751 + *sfp* by natural competence transformation^49^. The cloned gene clusters were integrated into the *amyE* gene loci of *B. subtilis* 1A751 + sfp by double crossover to obtain the final heterologous expression strains *B. subtilis* 1A751 + *sfp* + ede and *B. subtilis* 1A751 + *sfp* + bmy. To verify that the correct transformant resulting from double crossover, we selected 10 spectinomycin-resistant colonies from each transformation to conduct 5′ and 3′ junction colony PCR, as illustrated in Fig. 3A. According to the predicted size of the PCR products of both 5′ and 3′ junction PCR, all ten colonies from the transformation of plasmids p15A-amyEF-Spect-amyER-ede with both 5′ junction PCR fragments and 3′ junction PCR fragments showed the correct insertion resulting from double crossover event (Fig. 3B). Two colonies in the transformation of plasmid p15A-amyEF-Spect-amyER-bmy2 with both 5′ junction PCR fragments and 3′ junction PCR fragments showed the correct insertion resulting from double crossover, and other five colonies with only 3′ junction PCR fragments might have resulted from a single crossover event (Fig. 3C). The correct transformants were subsequently analysed using HPLC-MS to detect the products (Fig. 3D). Comparative analysis of the metabolite profiles between *B. subtilis* 1A751 + *sfp* and *B. subtilis* 1A751 + *sfp* + ede/bmy were performed using HPLC-MS. No obvious difference was detected between the metabolite profiles of *B. subtilis* 1A751 + *sfp* and *B. subtilis* 1A751 + *sfp* + ede (data not shown). However, three additional peaks were detected in *B. subtilis* 1A751 + *sfp* + bmy and were identified as bacillomycin D analogues, C14-bacillomycin D (m/z 1031.5), C15-bacillomycin D (m/z 1045.6) and C16-bacillomycin D (m/z 1059.6) (Fig. 3D/E). This result indicated that the *bmy* gene cluster from *B. amyloliquefaciens* FZB42 was successfully expressed in heterologous host *B. subtilis* 1A751 + *sfp*, which demonstrated that this two-step procedure of direct cloning and heterologous expression is effective and efficient. To examine the stability of the *bmy* gene clusters in the heterologous host, we monitored bacillomycin production and performed PCR to detect several different regions in the gene cluster after several rounds of culture in LB medium containing antibiotics. The results showed that the *bmy* gene cluster were stably maintained in the host(data not shown).

## Discussion

Red/ET recombineering has been widely applied for direct cloning and modification of natural product biosynthetic pathways^31^,^32^,^33^,^34^,^35^,^36^,^37^,^38^. Herein, we specially facilitate the application of Red/ET recombineering for direct cloning and heterologous expression of natural product biosynthesis gene clusters in *B. subtilis*. To clone and express a desired gene cluster in *B. subtilis*, we just need to synthesize a pair of primers containing 70-bp homologous arms to generate a linear cloning vector flanked with the homologous arms using PCR based on a specific cloning vector. After direct cloning by LLHR, the cloned gene clusters can be directly integrated into the chromosome of *B. subtilis* without any further modification. All of the procedures can be completed within one week if the gene cluster is intact and has no homologous region with the host strain.

Li *et al*. reported a similar strategy of rapid “plug-and-play” genomic capture and expression of bacilli-based natural products in *B. subtilis* via TAR^22^, which also uses a specific capture vector that contains elements for gene cluster horizontal transfer to *B. subtilis.* In contrast to this approach, there are three major advantages to the method present herein. First, it is based on Red/ET recombineering in *E. coli*, is simpler and more convenient for using short homologous arms (70 bp), omitting several steps of inserting two 1-kb capture arms corresponding to gene clusters into the cloning vector and of reintroducing vectors contain cloned gene cluster from *Saccharomyces cerevisiae* into *E. coli*. Second, the use of a shorter linear cloning vector (4.2 kb) coupled with a CcdB toxin protein gene dramatically decreases the rate of negative clones, reducing the likelihood of vector self-cyclization and decreasing the background of the original vector^31^,^48^. Third, compared with the unregulated yeast homologous recombinase in TAR, the recombinase system in Red/ET recombineering is strictly regulated by an arabinose-inducible promoter P_BAD_^31^, which reduced the occurrence of unintended recombination in the gene cluster after it has been cloned^29^.

The failure to achieve heterologous expression of the gene cluster for edeine in *B. subtilis*, likely reflects a mutation in the cloned gene cluster of edeine, as edeine shows strong antibiotic activity to both Gram-positive and Gram-negative bacteria^39^. Subsequently, the entire plasmid p15A-amyEF-Spect-amyER-ede (52.9 kb) which was selected to transform into *B.subtilis* was completely sequenced. However, the sequencing result showed that no mutation was detected in the gene cluster. Therefore, there might be other reasons that lack heterologous edeine expression. First, *B. subtilis* might not produce all of the necessary precursors required for edeine, such as spermidine and 1,3-Diaminopropane^39^. Second, the promoter of *ede* gene clusters from *Brevibacillus brevis* might be incompatible with *B. subtilis,* reflecting the comparatively distant phylogenetic relationship between these species. Further studies of edeine heterologous expression are underway to elucidate these hypothetical reasons.

In summary, we constructed a specific cloning vector for the direct cloning of biosynthetic gene clusters of natural products from bacilli and simplify further modifications after direct cloning. In addition, we successfully cloned two gene clusters using this vector and detected products of bacillimycin. These results demonstrate the significant potential use of Red/ET recombineering-mediated direct cloning and heterologous expression for genome mining of novel natural products or improving the yield of compounds from bacilli.

## Methods

### Strains, plasmids and culture conditions

The bacterial strains and plasmids used in the present study are shown in Table S1. All primers were synthesized at Sangon Biotech (Shanghai, China) Co., Ltd. (Table S2). All restriction enzymes and DNA markers were purchased from New England Biolabs (UK) Ltd.(Hertfordshire, UK). Taq polymerase was purchased from TaKaRa Bio Inc. (Dalian, China). DNA sequencing was performed at Hunan Tsingke Biotech Co., Ltd. (Changsha, China). *E. coli* and *B. subtilis* cells were cultured in Luria-Bertani (LB) liquid media or on LB agar (1.2% agar). For *E. coli*, chloramphenicol (15 μg mL^−1^), ampicillin (100 μg mL^−1^) or spectinomycin (60 μg mL^−1^) were added to the media as required. *B. subtilis* recombinants were selected on LB medium containing chloramphenicol (5 μg mL^−1^) and spectinomycin (80 μg mL^−1^).

### Repair of a point mutation in the *sfp* gene of *B. subtilis*

The dot mutation of the *sfp* gene was corrected using overlapping-extension PCR (fusion PCR), as illustrated in Fig. S1A. The correct *sfp* gene was introduced into the *B. subtilis* 1A751 chromosome via the integration vector pBR322-Amp-HAF-sfp-lox-cm-HAR. The integration vector was constructed from four gene fragments, pBR322-Amp, HAF-sfp, lox71-cm-lox66, and HAR, via Red/ET multiple recombination (Fig. S1B). The pBR322-Amp was PCR amplified from vector pBR322-Amp-U6-ccdB-cm using primers pBR322-Amp-F/R. HAF was amplified from the genomic DNA of *B. subtilis* 1A751 using primers yczE-R(P1)/sfp-correct-F(P2), and the sfp-R was amplified using primers sfp-correct-R(P3)/sfp-F(P4). The HAF-sfp was amplified from OE-PCR of HAF, and sfp-back was amplified using primers yczE-R(P1)/sfp-F(P4). The lox71-cm-lox66 was amplified from vector pAD123 using primers lox71-cm-F/lox66-cm-R. HAR was amplified from genomic DNA. Red/ET multiple recombineering was conducted according to a previous approach^38^. The correct recombinant vector was confirmed by restriction enzyme digestion and sequencing. The vector pBR322-Amp-HAF-sfp-lox-cm-HAR, containing 1.0-kb homology regions corresponding to the upstream and downstream adjacent regions of the gene *sfp*, were linearized and subsequently transferred into *B. subtilis* 1A751 by natural competence transformation. The correct *sfp-*repaired strains *B. subtilis* 1A751-sfp-lox-cm were screened by PCR, followed by sequencing and haemolysis testing^47^. After transformation and induction of the Cre-expression vector pDG148-Cre, the chloramphenicol resistance gene (Cm) was excised from the chromosome of *B. subtilis* 1A751-sfp-lox-cm, resulting in the host strain *B. subtilis* 1A751 + sfp (Fig. 1B). Natural competence transformation of *B. subtilis* was conducted according to Cutting and Vander^49^.

### Construction of the cloning vector for direct cloning of gene clusters

The cloning vector p15A-amyEF-Amp-ccdB-Spect-amyER, was constructed from the following five gene fragments, p15A-origin, amyEF, Amp-ccdB, Spect, and amyER via Red/ET multiple recombination, as illustrated in Fig. S2. The p15A-origin was PCR amplified from vector p15A-amp-ccdB using primers p15A-F/R. The 1-kb upstream and 1-kb downstream homologous arms of the *B. subtilis amy*E gene, namely amyEF and amyER, required for gene cluster integration in *B. subtilis*, were PCR amplified from *B. subtilis* 168 genomic DNA using primers amyEF F/R and amyER F/R, respectively. The Amp-ccdB fragment, comprising the Amp resistance gene and toxic protein CcdB gene, was PCR amplified from vector p15A-amp-ccdB using primers Amp-ccdB F/R. Spect, the spectinomycin resistance gene, was amplified from vector p7S6 using primers Spect-F/R. Five fragments with 50-bp overlapping sequences with the adjacent fragment at both terminal, were co-transformed into GB05dir-gyrA462^48^, a CcdB-resistant *E. coli* strain containing the mutation GyrA R462M mutation and LLHR-proficient recombinases (RecET, Redγ, and RecA). Red/ET multiple recombineering was conducted as previously described^38^. The correct recombinant vector was confirmed by restriction enzyme digestion and sequencing.

### Bacterial genomic DNA isolation

Bacterial genomic DNA isolation was performed as previously described with slight modifications^31^. *B. amyloliquefaciens* FZB42 or *Brevibacillus brevis* X23 was cultured overnight in 50 mL of LB medium at 30 °C. After centrifugation of 1.8 mL culture in a 2 ml centrifuge tube, and the cells were washed once with 1 mL of sterile water, and subsequently resuspended in 450 μL of 10 mM Tris-HCl, pH 8.0. After adding 22.5 μL of lysozyme (20 mg mL^−1^) at a final concentration of 1 mg mL^−1^ followed by incubation at 37 °C for 1 h, 30 μL of proteinase K (20 mg mL^−1^) and 40 μL of 10% SDS were added and mixed well by inverting the tubes several times. The mixture was incubated at 50 °C for 1~2 h with occasional inversion until the solution became clear. The solution was combined with 500 μL phenol: chloroform: isoamyl alcohol (25:24:1) and the tubes were inverted more than 30 times until the mixture was completely white. After centrifugation at 13500 rpm for 15 min, 300 μL of aqueous phase solution was transferred to a fresh 1.5 mL tube using a 1 mL pipette tip (the little end of tip was cut to create a larger opening), and the DNA was precipitated after adding 35 μL of 3 M NaOAc (pH7.5) and one volume of isopropanol, with gentle inverting. The DNA was transferred to a new 2-mL centrifuge tube, rinsed with 1 mL of 75% ethanol. The mixture was centrifuged for 1 min at 1,000 rpm, the supernatant was discarded, and the DNA pellet was dried on the bench for 15-25 min, and dissolved in 200 μL of ddH_2_O.

### Direct cloning of the *bmy* and *ede* gene clusters using Red/ET recombination

Direct cloning was performed as previously described with slight modifications^31^. To clone the *bmy* gene cluster, linear cloning vector p15A- amyEF-Spect-amyER, flanked with 70 bp homology arms to target the gene cluster was PCR amplified from vector p15A-amyEF-Amp-ccdB-Spect-amyER using primers bmy-F/R. Approximately 20 μg of the genomic DNA of *B. amyloliquefaciens* FZB42 was digested with CspCI, for 4~6 h and subsequently extracted with phenol: chloroform: isoamyl alcohol (25: 24: 1) to remove the residual restriction enzyme. After precipitation and washing, the digested genomic DNA (10 μg) was mixed with 200 ng of the linear cloning vector and co-transformed into *E. coli* GB05-dir, in which araC-P_BAD_-redγRecET was integrated into the chromosome by electroporation^31^. After plasmid extraction, the correct transformant containing the *bmy* gene cluster was confirmed by restriction analysis, resulting in p15A-amyEF-Spect-amyER-bmy. Using the same procedure, the genomic DNA of *Brevibacillus brevis* X23 was digested using *Swa*I and *Eag*I, and the *ede g*ene cluster was cloned using primers ede-F/R, resulting in an recombinant plasmid p15A-amyEF-Spect-amyER-ede containing *ede* gene cluster.

### Deletion of a redundant homologous region in p15A-amyEF-Spect-amyER-bmy

The plasmid p15A-amyEF-Spect-amyER-bmy was transferred into *E. coli* GB05-Red, in which araC-P_BAD_-redγβα was integrated into the chromosome^31^. The lox71-cm-lox66 fragment was PCR amplified from vector p15A-cm-ccdB^48^ using primers lox-cm-F/R, and subsequently was introduced into *E. coli* GB05-Red harboring p15A-amyEF-Spect-amyER-bmy. After recombination, lox71-cm-lox66 replaced the redundant genomic region of p15A-amyEF-Spect-amyER-bmy. The resulting plasmid p15A-amyEF-Spect-amyER-bmy-lox-cm was confirmed by restriction enzyme digestion analysis. After introducing and inducing the expression of a Cre plasmid pSC101-BAD-Cre-tet, the chloramphenicol resistance gene (Cm) was excised from p15A-amyEF-Spect-amyER-bmy-lox-cm to obtain p15A-amyEF-Spect-amyER-bmy2.

### Heterologous expression of the *bmy* gene cluster

The plasmid p15A-amyEF-Spect-amyER-bmy2 or p15A-amyEF-Spect-amyER-ede, flanked by two 1-kb homologous regions corresponding to the upstream and downstream regions of the gene *amy*E in *B. subtilis* 1A751 + *sfp*, were transferred into *B. subtilis* 1A751 + sfp by natural competence transformation to obtain *B. subtilis* 1A751 + *sfp* + bmy or *B. subtilis* 1A751 + *sfp* + ede. To detect the correct transformants of the *ede* gene cluster, spectinomycin-resistant clones were identified by 5′ and 3′ junction colony PCR using primers amyE-T-5/ede-T-5 and amyE-T-3/ede-T-3, respectively (Fig. 3A/B). To detect the correct transformant of the *ede* gene cluster, spectinomycin-resistant clones were identified by 5′ and 3′ junction colony PCR using primers amyE-T-5/bmy-T-5 and amyE-T-3/bmy-T-3, respectively (Fig. 3A/C). PCR-positive clones were routinely grown in LB broth containing spectinomycin (60 μg mL^−1^) at 30 °C overnight. 2% (1.0 mL) of the preculture was inoculated into 50 mL of BPY media containing spectinomycin (60 μg mL^−1^) and grown at 30 °C in a 250-mL flask with constant agitation at 180 rpm. After culture for 24 h, 2% sterilized resin Amberlite XAD-16 was added and the incubation was continued for two more days. After harvesting by centrifugation at 8,000 rpm for 8 min, the pellets and resins were extracted with 30 mL of methanol. The extracts were evaporated and dissolved in 1 mL of methanol and used for HPLC-MS analysis. The HPLC-MS measurement was performed on a Thermo Scientific Dionex Ultimate 3000 LC system coupled with a Bruker impact HD Q-TOF mass spectrometer. The MS data were acquired in the positive ion mode with a range of 200–2,000 m/z scans with auto MS2 fragmentation. Reversed-phase chromatography of UPLC was conducted with 250 × 4.6 mm, 5 μm particle size columns (YMC, C18, Japan) at a solvent gradient (with solvents A (water and 0.1% formic acid) and B (CH_3_CN and 0.1% formic acid): 5% B from 0 to 5 min, 5% B–95% B within 40 min, followed by 15 min with 95% B at a flow rate of 0.75 mL/min). The MS measurement was conducted on a Q-TOF mass spectrometer (Bruker Daltonics, Bremen, Germany) using a standard ESI source.

## Additional Information

**How to cite this article**: Liu, Q. *et al*. Simple and rapid direct cloning and heterologous expression of natural product biosynthetic gene cluster in *Bacillus subtilis* via Red/ET recombineering. *Sci. Rep.* **6**, 34623; doi: 10.1038/srep34623 (2016).

## Supplementary Material

Supplementary Information
