# Supplementary material for: Simple and rapid direct cloning and heterologous expression of natural product biosynthetic gene cluster in Bacillus subtilis via Red/ET recombineering
Source: Sci Rep. 2016 Sep 30;6:34623. doi: 10.1038/srep34623 (PMC5043344; doi:10.1038/srep34623)
Supplement: Supplementary Information [file srep34623-s1.pdf]

Supplementary Information

Simple and rapid direct cloning and heterologous expression of natural product biosynthetic gene cluster in *Bacillus subtilis* via Red/ET recombineering

Qingshu Liu[1,2,3], Qiyao Shen[2], Xiaoying Bian[2], Hanna Chen[2], Jun Fu[2], Hailong Wang[2], Ping Lei[3], Zhaohui Guo[3], Wu Chen[1, *], Dingjun Li [1,4,*], and Youming Zhang[2,*]

[1]College of Plant Protection, Hunan Agricultural University, Changsha, 410128, People's Republic of China,

[2]Shandong University–Helmholtz Institute of Biotechnology, State Key Laboratory of Microbial Technology, School of Life Science, Shandong University, Jinan, 250100, People's Republic of China,

[3]Hunan Institute of Microbiology, Changsha, 410009, People's Republic of China,

[4] Hunan Business College, Changsha, 410205, People's Republic of China

Table S1. Strains and plasmids used in this study.

Strain or plasmid	Characteristics	Sources
Strains		
E. coli GB05	F-*mcr*A Δ(*mrr-hsd*RMS-*mcr*BC) *φ*80*lac*ZΔM15 Δ*lac*X74 *rec*A1 *end*A1 *ara*D139 Δ(*ara*, *leu*)7697 *gal*U *gal*K λ *rpsLnupGfhu*A::IS2 *rec*ET *redα*, phage T1-resistent	31
E. coli GB05-dir	GB2005, *ara*C-BAD-ETγA	31
E. coli GB05-red	GB2005, *ara*C-BAD-γβαA	31
E. coli GB05dir-gyrA462	GB05dir, *gyr*A462 (*ccd*B resistant)	47
B.subtilis 1A751	*his nprR*2 *nprE*18 △*aprA*3 △*eglS*102 △bglT *bglS*RV	BGSC
B.amyloliquefaciens FZB42	bacillomycin-producing wild-type strain	BGSC
Brevibacillus brevis X23	edeine producer strain	40
B.subtilis 1A751+sfp	*B.subtilis* 1A751, repaired the mutated sfp gene	This study
B.subtilis 1A751+sfp+bmy	bacillomycin gene cluster in *B.subtilis* 1A751-sfp+,SpectR	This study
B.subtilis 1A751+sfp+ede	edeine gene cluster in *B.subtilis* 1A751-sfp+,SpectR	This study
Plasmid		
p15A-Amp-ccdB	p15a origin, ampR	47
p7S6	puc origin, ampR, SpecR	48
p15A-amyEF-Amp-ccdB -Spect-amyER	direct cloning vector, p15a origin, ampR, SpectR, with amyE homologous arm	This study
p15A-amyEF-Spect-amyER-ede	p15A replicon, containing genomic region of the entire edeine gene cluster, SpectR, with amyE homologous arm	This study
p15A-amyEF-Spect-amyER-bmy	p15A replicon, containing genomic region of entire bacillomycin gene cluster, SpectR, with amyE homologous arm	This study
p15A-amyEF-Spect-amyER-bmy -lox71-Cm-lox66	p15A replicon, delete the homologous region between host and producing stain in the gene cluster ,SpecR,lox71-CmR-lox66	This study
pSC101-BAD-Cre-tet	Cre expression vector in E.coli, pSC101 replicon, pBAD promoter, tetR	Lab stores
p15A-amyEF-Spect-amyER-bmy2	p15A replicon, delete the homologous region between host and producing stain in the gene cluster ,SpecR,lox72	This study

Table S2. Oligonucleotides used in this work.

Oligonucleotide	(5'-3') sequence
pBR322-Amp-F	AATTTTCGGCCAAGCTCTCGTAAACAATGACGTGAATCCGACGATGAG**GGATCCGCTA**GCGCTCTTCCGCTTCCTCGCT
pBR322-Amp-R	ATAAACAAATCGTTTTTGTATGTATCAATCGCTTTGTTGATGCAGTGCTGAAGGGCGAATTCTGCAGATATCC
yczE-R(P1)	CTCATCGTCGGATTCACGTCAT
sfp-correct-F(P2)	AGCGATAAGCCTTTGCCTTCCTGTTTGATAAAGCTTTCTTTCAT
sfp-correct-R(P3)	ATGAAAGAAAGCTTTATCAAACAGGAAGGCAAAGGCTTATCGCT
sfp-F (P4)	GGTTTTCTAATGTCACATAACTTCGTATAGCATACATTATACGAACGGTAGCCATTTATAAAAGCTCTTCGTAC
lox71-cm-F	TACCGTTCGTATAGCATACATTATACGAAGTTATTCCTTTAAAAAAACACAAAAGACCACA
lox66-cm-R	TACCGTTCGTATAATGTATGCTATACGAAGTTATGTGACATTAGAAAACCGACTGTAAAAAGT
ycxD-F	CAGCACTGCATCAACAAAGCGA
ycxD-R	GTGTTTTTTTAAAGGAATAACTTCGTATAATGTATGCTATACGAACGGTAGCTTTTATAAATGGCTCATCAACAGC
p15A-F	ACAACTTATATCGTATGGGGCT
p15A-R	CGGAGTGTATACTGGCTTACTA
amyEF-F	TTCACTGACACCCTCATCAGTGCCAACATAGTAAGCCAGTATACACTCCGCTCCAGTCTTCACATCGGTTTG
amyEF-R	TGAGCGGATACATATTTGAATGTATTTAGAAAAATAAACAAAGCGCTAGCTCTTCATCATCATTGGCATACG
Amp-ccdB-F	GCTAGCGCTTTGTTTATTTTTCTA
Amp-ccdB-R	TTTGTTCAAAAAAAAGCCCGCTCA
Spect-F	TGGGGAATATAACCCAGCCCGCCTAATGAGCGGGCTTTTTTTTGAACAAA**GGATCCCAGCTG**CGATTTTCGTTCGTGAATACATG
Spect-R	GGCGTACTGCCTGAACGAGAAGCTATCACCGCCCAGCCTAAACGGATATCGCTATACGAAGTTATTTATAAT
amyER-F	GATATCCGTTTAGGCTGGGCG
amyER-R	TATCTCTTCAAATGTAGCACCTGAAGTCAGCCCCATACGATATAAGTTGT**AAGCTT**CAATGGGGAAGAGAACCGCTT
bmy-F	AATGTATTGATTGCTTCTGTATACGCTGCTGACATCGTGCTTAAAGCGATGCCCATTCCTCCTGATGCTCTTCATCATCATTGGCATAC
bmy-R	CCGGGAGTTACTGAAGTTTCAGCAGCAAGTGATGCAACAGTTAATATATCTGCAGAAAGACAAGTGATCGAATGGCGATTTTCGTTCGTG
lox-cm-F	TTCATATGTCGTTTATGGACTTAGAAAACGTACCTTTGTCTCCCTTTGGCAAGGGCTGCTAAAGGAAGCG
lox-cm-R	CGGAGTCTGCTTTTTACTGCCGGTACTGCAATTTGTTCCCAAATGGTAGGGCAGGATAGGTGAAGTAGG
amyE-T-5	GAATGGGCTGCAAGCCTTGTG

amyE-T-3	TCGGTAAGTCCCGTCTAGCCT
bmy-T-5	TCTGCGAGCGTTCCTTGGGG
bmy-T-3	GGGAACAATCATCATGCTGT
ede-F	CTGGTCTAATCCTGTCAGTGTAAAATGAGGAGCCGCAAAGCCAACTTCCGGTTTTTGAACCTCTGCTACGTCTTCATCATCATTGGCATACG
ede-R	CAACGAAAAGAATTCGAAGAAGACATGGAAAGAAGCTGCTCTGACTATCGACATAAGAAATCAGCGGGCCCGAATGGCGATTTTCGTTCGTG
ede-T-5	GCAACTGGCGCAGTTCTAGCT
ede-T-3	CGTAGGGCCGCTGGACTTCT

Underlined sequences indicate homology arms.
Bold letters represent restriction enzyme sites.

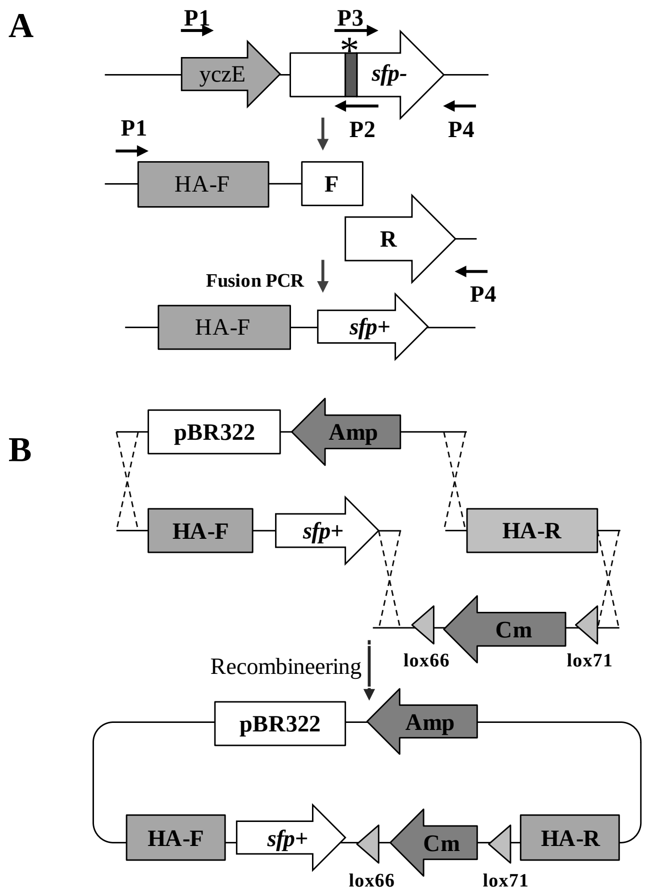

Figure S1. Scheme of repairing the insertional mutation of *sfp* gene in *B.subtilis* 1A751. (A) Deletion of the insertional mutation in *sfp* gene of *B.subtilis* 1A751 by fusion PCR. Fisrt, the front fragment was PCR amplified by Primer P1/P2, and the back fragment was amplified by primer P3/P4. Then two PCR products was assembled by fusion PCR. (B) Construction of a integraion vector for knock-in the repaired sfp gene by Red/ET quadruple homologous recombination. Four PCR fragments were obtained by PCR.

.

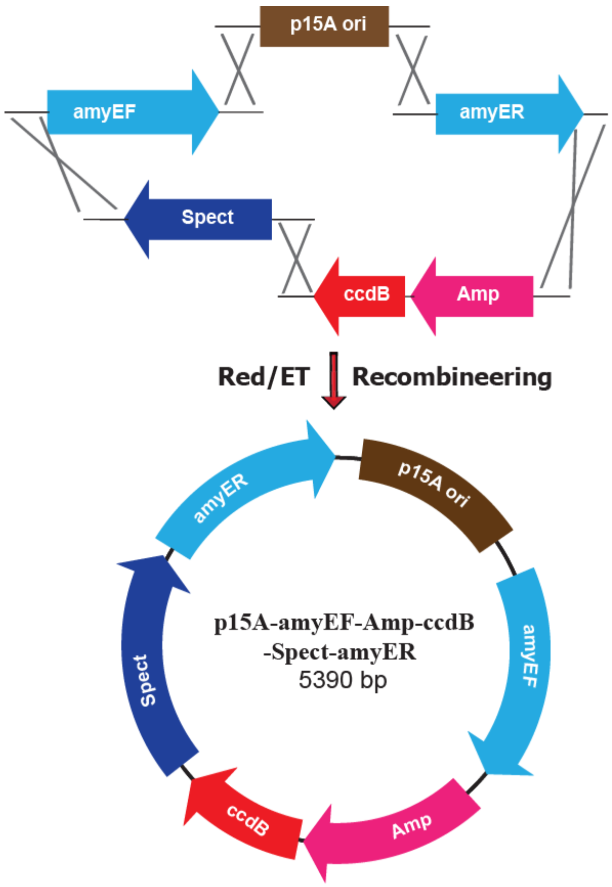

Figure S2. Quintuple recombineering to construct the vector for direct cloning and heterologous expression of natural product biosynthesis gene cluster in Bacillus subtilis. Five fragments (p15A-ori, amyEF, Amp-ccdB, Spect, and amyER) were obtained by PCR. An ampicillin resistance gene and ccdB were co-expression under the same promoter.

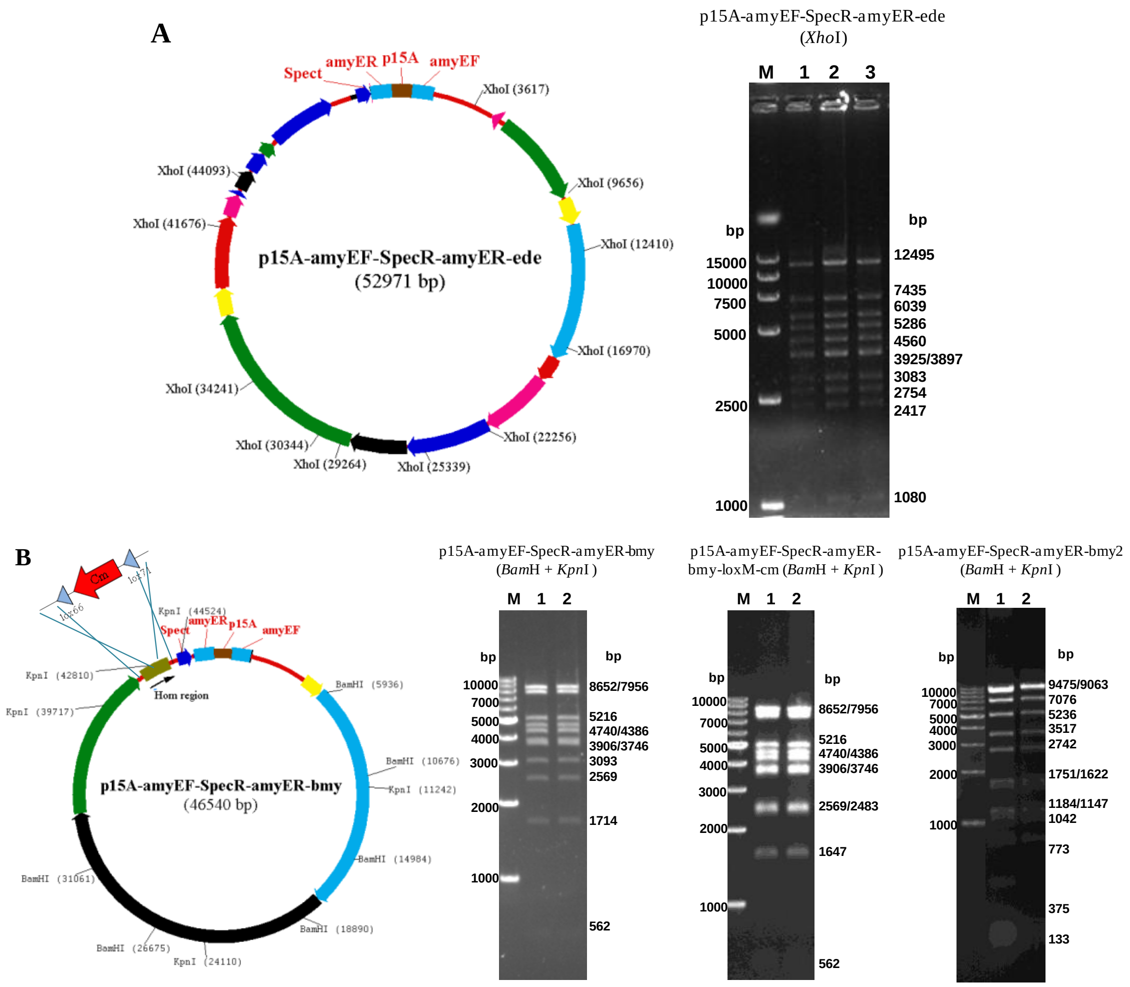

Figure S3 Restriction mappings of p15A-amyEF-Spect-amyER-ede, p15A-amyEF-Spect-amyER-bmy and it's derivatives. (A) Physical map and digestion spectra of p15A-amyEF-Spect-amyER-ede (*Xho*I). Lane M is the Takara DL 15000 DNA marker. Lanes 1–3 are correct recombinants. (B) Physical map and digestion spectra of p15A-amyEF-Spect-amyER-bmy and it's derivatives. (*Bam*HI+*Kpn*I). Lane M is the Takara 1kb ladder. Lane Lanes 1–3 are correct recombinants. The 1.5 kb homolgous (Hom) region were knocked-out with the lox71-cm-lox66 cassette via LCHR mediated by Redαβγ in *E. coli* GB08-Red, resulting in construct of p15A-amyEF-Spect-amyER-bmy-loxM-cm. After induction a Cre-expression plamid pSC101-BAD-Cre-Tet, the cm antibiotic resistance gene was excised, resulting in construct p15A-amyEF-Spect-amyER-bmy2.

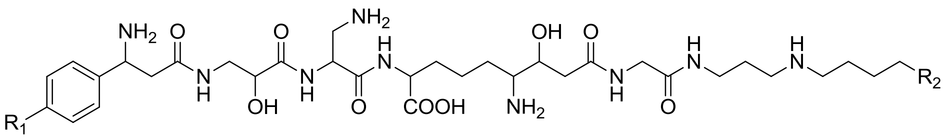

edeine

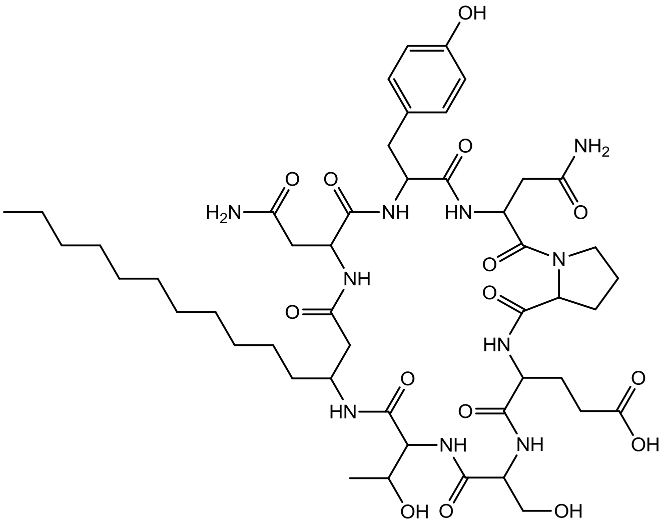

bacillomycin

Figure S4 Chemical structures of edeine and bacillomycin
